# Non-persistent exposures from plasticizers or plastic constituents in remote Arctic communities: a case for further research

**DOI:** 10.1038/s41370-022-00425-w

**Published:** 2022-03-28

**Authors:** Amira Aker, Élyse Caron-Beaudoin, Pierre Ayotte, Sylvie Ricard, Véronique Gilbert, Ellen Avard, Mélanie Lemire

**Affiliations:** 1 Axe santé des populations et pratiques optimales en santé, Centre de recherche du CHU de Québec-Université Laval, 1050 Ch Ste-Foy, Quebec, QC Canada; 2 Département de médecine sociale et préventive, Université Laval, 1030 Avenue de la Médecine, Quebec, QC Canada; 3 Department of Health and Society, University of Toronto Scarborough, 1265 Military Trail, Toronto, ON Canada; 4 Centre for Clinical Epidemiology and Evaluation, University of British Columbia, Vancouver Coastal Health Research Institute, 828 West 10th Avenue, Research Pavilion, Vancouver, BC Canada; 5 Centre de Toxicologie du Québec, Institut National de Santé Publique du Québec, Quebec, QC Canada; 6 Nunavik Regional Board of Health and Social Services, Kuujjuaq, QC Canada; 7 Kativik Regional Government based in Saint-Laurent, Saint-Laurent, QC Canada; 8 Nunavik Research Centre, Makivik Corporation, Kuujjuaq, QC Canada; 9 Institut de Biologie Intégrative et des Systèmes, Université Laval, 1030 Avenue de la Médecine, Quebec, QC Canada

**Keywords:** Non-persistent, Arctic, Exposure distribution, Phthalates, Bisphenols, Alternative plasticizers

## Abstract

**Background:**

Persistent organic pollutant exposures are well-documented in the Arctic, but fewer studies examined non-persistent chemicals, despite increased market food and consumer product consumption.

**Objective:**

To measure phenol, paraben, phthalate, and alternative plasticizer concentrations in Inuit adults.

**Methods:**

The study included 30 pooled urine samples from Qanuilirpitaa? 2017 Nunavik Inuit Health Survey (Q2017) participants. Creatinine-adjusted geometric mean concentrations (GM) and 95% confidence intervals (CI) were compared across sex, age, and regions, and compared to those in the Canadian Health Measures Survey (CHMS) and the First Nations Biomonitoring Initiative (FNBI).

**Results:**

Q2017 bisphenol-A concentrations were double the CHMS 2018–2019 concentrations [GM (95% CI): 1.98 (1.69–2.31) versus 0.71 (0.60–0.84) µg/g creatinine], but in line with FNBI [1.74 (1.41–2.13) µg/g creatinine]. Several phthalate concentrations were higher in Q2017 versus CHMS, particularly monobenzyl phthalate, which was was 19-fold higher in Q2017 versus CHMS 2018–2019 [45.26 (39.35–52.06) versus 2.4 (2.0–2.9) µg/g creatinine] and four-fold higher than FNBI. There were also four-fold higher concentrations of the two alternate plasticizer 2,2,4-trimethyl-1,3-pentanediol diisobutyrate (TIXB) metabolites in Q2017 compared to CHMS 2018–2019. Women and people living in Ungava Bay had generally higher concentrations of non-persistent chemicals.

**Significance:**

The results suggest higher concentrations of certain non-persistent chemicals in Inuit versus the general Canadian population.

**Impact:**

Few studies have explored non-persistent chemical distributions in Northern communities, despite the increasing consumer product and market food consumption. We analyzed 30 pooled samples from the Qanuilirpitaa? Nunavik Inuit Health Survey 2017 to assess exposures to common plasticizes and plastic constituents and compare their levels with the general Canadian population and First Nation groups. We observed particularly higher levels of bisphenol-A, of monobenzyl phthalate, and of two 2,2,4-trimethyl-1,3-pentanediol diisobutyrate (TXIB) metabolites among Nunavimmiut compared to the general Canadian population, notably among women and Ungava Bay residents. Larger studies are required to confirm our findings and identify potential adverse health effects from these exposures.

## Abstract (web summary)

Geometric means of select non-persistent chemicals in Nunavik, CHMS, and FNBI

## Graphical abstract

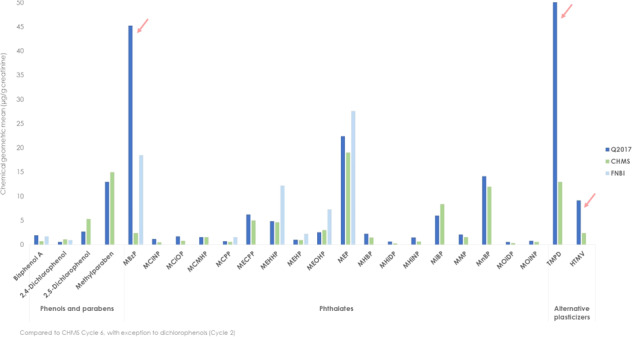

## Introduction

Northern communities have long been vulnerable to persistent organic pollutants and mercury that travel from southern latitudes and accumulate in local country foods [1]. As such, several studies have documented these exposures in Arctic wildlife and Inuit communities, and their health impacts [1–4]. However, to date, fewer studies examined exposure to non-persistent chemicals in these communities, and no biomonitoring initiatives in Nunavik, Canada included non-persistent chemicals despite their various possible exposure sources [1].

Phenols, parabens, and phthalates are examples of non-persistent chemicals, and are common food packaging and personal care product additives, as well as plastic additives or constituents [1, 5–8]. Additionally, alternative plasticizers, such as di(isononyl)cyclohexane-1,2-dicarboxylate (DINCH) and 2,2,4-trimethyl-1,3-pentanediol diisobutyrate (TXIB), are commonly used to replace phthalates. DINCH and TXIB are plasticizers used to increase the flexibility of products, and are regularly used in flooring, food packaging, and children’s products made with polyvinyl chloride [9–12].

There is a shift toward market food consumption at the expense of traditional country foods gathered from the local environment, and an increased use of consumer products in the North [13]. Market foods are routinely stored in cans and plastic packaging for months pre-purchase, and contaminants leaching into foods may result in elevated exposures [14, 15]. Furthermore, the combined effect of the melting permafrost and increasing landfill sizes with the growing Inuit population exacerbates the concern of non-persistent chemical exposure from landfill leachate [16, 17]. As such, there are growing concerns on the exposure and potential health impacts related to non-persistent chemicals in Arctic communities [1, 18, 19]. This is compounded with concerns regarding the low socioeconomic status, overcrowding, and food insecurity issues in Nunavik compared to the general Canadian population [1].

Many non-persistent chemicals are suspected endocrine disruptors, and have been associated with reproductive, neurodevelopmental, and cardiometabolic adverse effects [20, 21]. In other Arctic communities, exposure to phthalates were associated with lower birth weight in neonates [22], breast cancer in women [23], and adverse reproductive health outcomes in men [24, 25]. Studies exploring the health impacts of DINCH and TXIB are lacking, but animal studies suggest deleterious effects on the liver, kidney, thyroid and mammary glands with exposure to DINCH [26]. TXIB toxicity studies are limited; however, the liver (and potentially the kidneys) appear to be target organs in subchronic rat studies [27]. Another study found that TXIB has higher migration rates from products compared to other phthalates or plasticizers [27, 28], which may lead to elevated internal doses of TXIB versus other plasticizers.

Our objective was to explore biomarker concentrations of phenols (i.e., bisphenols, chlorophenols and triclosan), parabens, phthalates, and alternative plasticizers (i.e., DINCH and TXIB) in pooled samples from the Inuit population in Nunavik, Canada. We aimed to compare levels to those in the adult general Canadian population using the Canadian Health Measures survey (CHMS) and First Nation communities in Canada using the First Nations Biomonitoring Initiative (FNBI) where possible, compare chemical concentrations across sex, age, and ecological regions, and identify priority chemicals that warrant further analysis.

## Methods

**Fig. 1:**
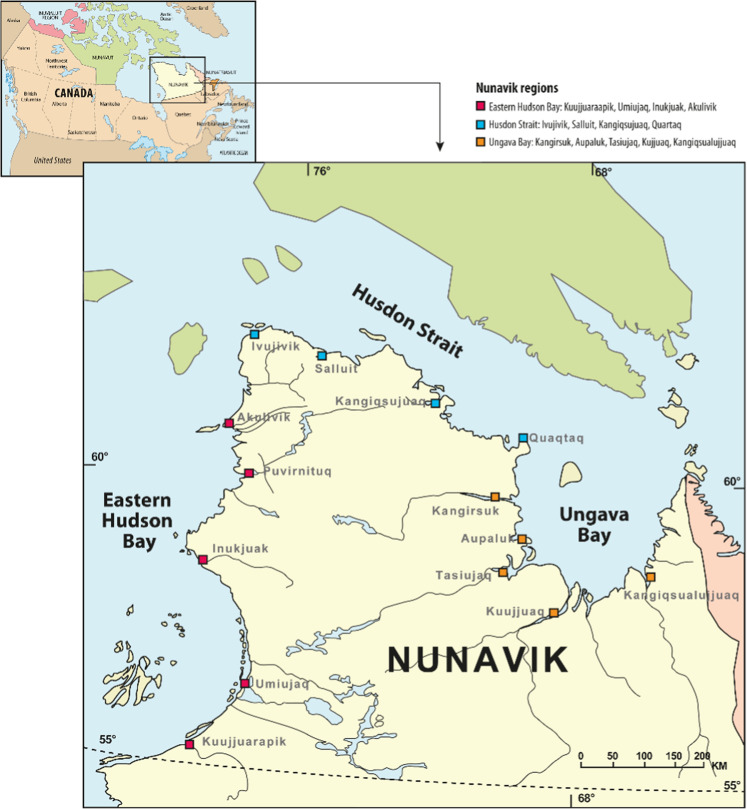
Map of Nunavik and its ecological regions (Hudson Bay, Hudson Strait, and Ungava Bay).

The Qanuilirpitaa? Nunavik Inuit Health Survey 2017 (Q2017) was conducted in the 14 Nunavik communities among Inuit aged 16 and over to provide a portrait of the population’s health status (Fig. 1). The survey used a stratified proportional model to select respondents based on community and age groups. This survey received ethical approval by the Comité d’éthique de la recherche du Centre Hospitalier Universitaire de Québec—Université Laval and was conducted in close collaboration with several Nunavik organisations and governed by the OCAP® principles (Ownership, Control, Access, and Possession).

A total of 1326 individuals participated in the data collection onboard the Amundsen, a Canadian Coast Guard Icebreaker, from August 19, 2017 to October 5, 2017. Data collection included questionnaires, biomarker sampling, and clinical measurements. Further details on the data collection, processing, and data analysis can be found in the Qanuilirpitaa? 2017 Methodological Report [29]. The overall response rate was 31% for those aged 16–30 years and 42% for people aged 31 years and over. The relatively lower response rate was largely due to high non-contact rates. Weighting was used to ensure population representativeness.

### Urine collection

Participants were instructed to collect their urine into a 60-ml polypropylene plastic jar and to tightly screw the cap on. The jar containing the urine sample was kept at room temperature until it was transported to the laboratory onboard the ship. Laboratory personnel transferred 1.5-ml aliquots of urine into 2-ml polypropylene tubes which were stored at −80 °C on board the Amundsen.

### Urine samples pooling strategy

Of the 1266 participants with urine samples, 30 pooled samples based on sex, age, and ecological region were analyzed for non-persistent chemicals to be cost-effective and ensure sufficient sample volumes for several contaminant measurement. Pools had a total volume of 15 ml of urine and were made by adding equal amounts of urine from a varying number of participants (see Table S1) that were grouped according to: sex (male and female); age (5 age groups :16–19 years; 20–29 years; 30–39 years; 40–59 years; 60+); and region of residence (Hudson Bay; Hudson Strait; Ungava Bay) (Table 1). Thus, these pooled samples are reflective of the population of Nunavik aged 16 and older in 2017.

**Table 1:** Geometric means of urine concentrations (µg/g creatinine) in 30 pooled samples created from the 1266 individuals recruited in Q2017 compared to Canadian Health Measures Survey and First Nations Biomonitoring Initiative.

	Q2017		CHMS		FNBI	
Chemical	%≥LOD	GM (95% CI)	%≥LOD	GM (95% CI)	%≥LOD	GM (95% CI)
Phenols						
Bisphenol A^a^	100	1.98 (1.69–2.31)	76.64	0.71 (0.60–0.84)	95.1	1.74 (1.41–2.13)
Bisphenol E	100	0.03 (0.03–0.04)	–		–	
Bisphenol F	100	0.82 (0.50–1.35)	–		–	
Bisphenol S	100	1.53 (1.23–1.91)	–		–	
Bisphenol Z	96.1	0.03 (0.03–0.03)	–		–	
4,4’-Biphenol	100	1.25 (1.12–1.40)	–		–	
2,4-dichlorophenol^b^	93.5	0.55 (0.43–0.70)	88.0	1.10 (0.91–1.20)	76.6	0.95 (0.63–1.43)
2,5-dichlorophenol^b^	100	2.71 (1.86–3.93)	95.9	5.30 (4.30–6.60)	–	
Triclosan^c^	89.1	20.18^d^ (13.01–31.31)	55.4	–^e^	–	
Parabens						
Ethylparaben^a^	60.6	1.22 (0.72–2.05)	36.1	–^e^	–	
Methylparaben^a^	95.3	10.65 (5.85–19.39)	84.8	13 (11–16)	–	
Propylparaben^a^	79.9	–^f^	60.6	1.4 (1.2–1.7)	–	
Phthalates						
MBzP^a^	100	45.26 (39.35–52.06)	98.2	2.4 (2.0–2.9)	100	18.58 (15.30–22.55)
MCiNP^a^	100	1.16 (0.86–1.55)	92.5	0.47 (0.41–0.54)	–	
MCiOP^a^	100	1.68 (1.28–2.22)	87.6	0.79 (0.70–0.90)	–	
MCMHP^a^	100	1.55 (1.05–2.28)	93.7	1.6 (1.5–1.8)	–	
MCPP^a^	100	0.69 (0.55–0.87)	84.8	0.57 (0.51–0.63)	96.9	1.58 (1.27–1.98)
MECPP^a^	100	6.24 (4.48–8.69)	99.6	5.0 (4.4–5.7)	–	
MEHHP^a^	100	4.83 (3.67–6.37)	99.9	4.6 (4.1–5.2)	100	12.26 (11.60–12.95)
MEHP^a^	100	1.04 (0.85–1.28)	98.8	0.94 (0.83–1.1)	98.8	2.27 (2.10–2.44)
MEOHP^a^	100	2.57 (1.92–3.44)	99.6	3.0 (2.6–3.4)	100	7.29 (6.87–7.74)
MEP^a^	100	22.44 (14.75–34.12)	99.4	19 (16–22)	100	27.67 (21.19–36.15)
MHBP^a^	100	2.27 (2.05–2.52)	98.6	1.5 (1.3–1.7)	–	
MHiDP^a^	100	0.62 (0.54–0.71)	73.1	0.26 (0.23–0.30)	–	
MHiNP^a^	100	1.45 (1.09–1.92)	84.6	0.63 (0.53–0.76)	–	
MiBP^a^	100	6.03 (5.47–6.64)	99.4	8.4 (7.7–9.2)	–	
MiNP^a^	61.4	0.31 (0.23–0.41)	46.9	–^e^	1.0	–^e^
MMP^a^	100	2.08 (1.74–2.48)	92.7	1.6 (1.4–1.7)	38.7	–^e^
MnBP^a^	100	14.11 (12.69–15.70)	99.7	12 (10–13)	–	
MOiDP^a^	100	0.54 (0.48–0.61)	73.9	0.33 (0.29–0.38)	–	
MOiNP^a^	100	0.83 (0.63–1.08)	83.8	0.56 (0.47–0.66)	–	
2-OH-MiBP	100	3.16 (2.82–3.53)	–		–	
Alternative plasticizers						
TMPD^a^	100	52.33 (45.41–60.32)	97.1	13 (11–16)	–	
HTMV^a^	100	9.16 (7.72–10.87)	95.5	2.4 (2.1–2.9)	–	
OH-MINCH^a^	93.2	0.13 (0.11–0.16)	47.2	–^e^	–	
oxo-MINCH^a^	81.8	0.04 (0.03–0.05)	34.6	–^e^	–	
* cis-cx-*MINCH^a^	63.9	0.03 (0.03–0.04)	25.3	–^e^	–	

Phthalates: *MBzP* monobenzyl phthalate, *MCiNP* mono(carboxy-isononyl) phthalate, *MCiOP* mono-(carboxy-isooctyl) phthalate, *MCMHP* mono-(2-carboxy-methylhexyl) phthalate, *MCPP* mono-3-carboxypropyl phthalate, *MECPP* mono-(2-ethyl-5-carboxypentyl) phthalate, *MEHHP* mono-(2-ethyl-5-hydroxyhexyl) phthalate, *MEHP* monoethylhexyl phthalate, *MEOHP* mono-(2-ethyl-5-oxohexyl) phthalate, *MEP* monoethyl phthalate, *MHBP* mono-(3-hydroxy-n-butyl) phthalate, *MHiDP* mono(hydroxy-isodecyl) phthalate, *MHiNP* mono(hydroxyl-isononyl) phthalate, *MiBP* mono-isobutyl phthalate, *MiNP* monoisononyl phthalate, *MMP* monomethyl phthalate, *MnBP* mono-n-butyl phthalate, *MOiDP* monooxoisodecyl phthalate, *MOiNP* monooxoisononyl phthalate, *2-OH-MiBP* mono-2-hydroxy-isobutyl phthalate. Alternative plasticizers: *TMPD* 2,2,4-Trimethyl-1,3-pentanediol, *HTMV* 2,2,4-Trimethyl-3-hydroxyvaleric acid, *OH-MINCH* 1,2-(Cyclohexanedicarboxylate)-mono-(7-hydroxy-4-methyl) octyl ester, *oxo-MINCH* 1,2-(Cyclohexanedicarboxylate)-mono-(7-oxo-4-methyl) octyl ester, *cis-cx-MINCH* 1,2-(cis-Cyclohexanedicarboxylate)-mono-(7-carboxylate-4-methyl) heptyl ester.

^a^Compared to CHMS Cycle 6 2018–2019 aged 16–79 years.

^b^Compared to CHMS Cycle 2 2009–2011 aged 20–79 years.

^c^Compared to CHMS Cycle 4 2014–2015 aged 20–79 years.

^d^Use data with caution. The coefficient of variation is between 16.6 and 33.3%.

^e^Geometric mean not reported if <60% of samples are below the LOD.

^f^Data are too unreliable to be published. The coefficient of variation is greater than 33.3%.

### Pooled sample preparation

Individual samples were thawed at room temperature and vortex mixed prior to pooling. The volume pipetted from each individual sample was equal to the volume of the pooled sample (15 ml) divided by the total number of participants in the subgroup. Pooled samples were prepared in 60-ml polypropylene containers and kept frozen at −20 °C until time of analysis. Two laboratory blanks consisting of polypropylene containers filled with ultrapure water were prepared at the same time as the pools and stored in the same conditions.

### Non-persistent chemical analyses

All laboratory analyses were completed at the Centre de toxicologie du Québec facilities of the Institut national de santé publique du Québec (INSPQ).

Urinary chlorophenols (2,4-dichlorophenol, 2,5-dichlorophenol), bisphenol-A (BPA), BPA analogs (bisphenols- AF, AP, B, CII, E, F, P, S, Z and 4,4’), and triclosan were hydrolyzed with a β-glucuronidase enzyme. After the samples were derivatized with pentafluorobenzyl bromide at 70 °C for 2 h, the products were extracted with a mixture of dichloromethane-hexane. Evaporated extracts were redissolved and analyzed by gas chromatography on an Agilent 6890 or 7890 coupled to a Waters Quattro Micro GC tandem mass spectrometer (MassLynx software) operating in multiple reaction monitoring (MRM) mode following a negative ion chemical ionization (INSPQ method E-454).

BPA, BPA analogs (bisphenols- AF, AP, B, CII, E, F, P, S, Z and 4,4’), and triclosan samples were adjusted to an alkaline pH and were then derivatized with pentafluorobenzyl bromide at 70 °C for 20 min. The derivatized products were extracted using SPE cartridges. Evaporated extracts were analyzed using a Waters Acquity UPLC coupled to a Waters Xevo TQ-S tandem mass spectrometer. The mass spectrometer was operated in the positive ion mode and the analytes were quantified using MRM (INSPQ method E-505). Free and conjugated forms of bisphenols and triclosan were measured together by this procedure.

For the analysis of benzyl paraben, butyl paraben, ethyl paraben, isobutyl paraben, methyl paraben, and n-Propylparaben, urine samples were subjected to an enzymatic hydrolysis (β-glucuronidase enzyme). The samples were then acidified and extracted with chlorobutane (liquid-liquid extraction). Evaporated extracts were measured using a Waters Acquity UPLC coupled to a Waters Xevo TQ-S tandem mass spectrometer operating in MRM mode following an electrospray ionization in negative ion mode (INSPQ method E-474).

A total of 24 phthalate metabolites were measured [monobenzyl phthalate (MBzP), monocyclohexyl phthalate (MCHP), mono-(7-carboxy-n-heptyl) phthalate (MCHpP), mono(carboxy-isononyl) (MCiNP), mono(carboxyisooctyl) phthalate (MCiOP), mono(2-carboxymethylhexyl) phthalate (MCMHP), mono-(3-carboxypropyl) phthalate (MCPP), mono(2-ethyl-5-carboxypentyl) phthalate (MECPP), mono(2-ethyl-5-hydroxyhexyl) phthalate (MEHHP), mono-(2-ethyl-5-hexyl) phthalate (MEHP), mono-(2-ethyl-5-oxohexyl) phthalate (MEOHP), monoethyl phthalate (MEP), mono-3-hydroxybutyl phthalate (MHBP), monohydroxyisodecyl phthalate (MHiDP), mono(hydroxyisononyl) phthalate (MHiNP), monoisobutyl phthalate (MiBP), mono-isodecyl phthalate (MiDP), monoisononyl phthalate, mono-methyl phthalate (MMP), mono-n-butyl phthalate (MnBP), mono-n-octyl phthalate (MnOP), monooxoisodecyl phthalate (MOiDP), mono(oxo-isononyl) phthalate (MOiNP), mono-hydroxy-isobutyl phthalate (2-OH-MiBP)]. Urine samples were hydrolyzed at 37 °C for 75 min with β-glucuronidase enzymatic solution in an ammonium acetate buffer at a pH of 6.5. The samples were acidified with phosphoric acid and extracted using a mixture of hexane-ethyl acetate (liquid-liquid extraction). The extracts were evaporated to dryness, reconstituted in an appropriate solvent and analyzed on a Waters Acquity UPLC coupled to a Waters Xevo TQ-S tandem mass spectrometer operating in MRM mode following an electrospray ionization in negative ion mode (INSPQ method E-490).

Two TXIB metabolites [2,2,4-trimethylpentane-1,3-diol (TMPD), 2,2,4-trimethyl-3-hydroxyvaleric acid (HTMV)] and one DINCH metabolite [(*cis*−1,4-cyclohexanedicarboxylic acid (CHDA)] were measured by UPLC-MS/MS using the INSPQ method E-497. Other alternative plasticizers, namely DINCH metabolites [cyclohexane-1,2-dicarboxylic acid (*cis*-CHDA), 1,2-(*trans*-cyclohexane-dicarboxylate)-mono-4-methyloctyl ester (*trans*-MINCH), 1,2-(cyclohexanedicarboxylate)-mono-(7-hydroxy-4-methyl) octyl ester (OH-MINCH), 1,2-(*trans*-cyclohexane-dicarboxylate)-mono-(7-carboxylate-4-methyl)heptyl ester (*trans*-cx-MINCH), 1,2-(*cis*-cyclohexane-dicarboxylate)-mono-(7-carboxylate-4-methyl) heptyl ester (*cis*-cx-MINCH)] as well as trioctyl trimellitate (TOTM) metabolites [1,2,4-benzenetricarboxylate 1-(2-ethylhexyl) ester (1-MEHTM), 1,2,4-benzenetricarboxylate 2-(2-ethylhexyl) ester (2-MEHTM) and 1,2,4-benzenetricarboxylate 4-(2-ethylhexyl) ester (4-MEHTM)] were measured by UPLC-MS/MS using the INSPQ method E-496.

Creatinine was measured in urine samples to account for urinary dilution. Creatinine was measured in urine using the colorimetric end-point Jaffe method. An alkaline solution of sodium picrate reacts with creatinine in urine to form a red Janovski complex. The absorbance was read at 510 nm on a Indiko Plus autoanalyzer (INSPQ method C-601).

All chemical limits of detection (LOD) can be found in Table S3.

### Statistical analysis

First, the percent of the pooled samples with levels equal to or above a chemical’s LOD in 1) the overall pooled samples, and 2) in each pooled sample strata were assessed. If ≥60% of the samples were at or above the LOD of any given chemical then further descriptive analyses were conducted for that chemical [the geometric mean (GM), 95% confidence intervals (CI)]. Otherwise, descriptive analyses were considered unreliable and not conducted. For descriptive analyses, pooled sample concentrations below the LOD were attributed a value of LOD/2. All measurements were adjusted for creatinine to account for urine dilution.

Pooled sample results were further weighted using survey weights calculated using sex, age, and ecological region community distribution of the underlying Nunavik population in 2017. These survey weights were assigned to every individual to indicate how much influence the individual could provide in the data analysis; thus, better representing population-level estimates. GMs, their associated 95% CIs, and coefficients of variation (CVs) were calculated using the sum of the survey weights of the individuals forming each pool.

If the CV was below 33%, chemical concentrations were compared between Q2017 and the most available information from CHMS [30–32] and FNBI (2011) [33]. Population GMs were considered significantly different if the 95% CI of the GMs did not overlap. CHMS Cycles 2 (2009–2011) [31], 4 (2014–2015) [32], and 6 (2018–2019) [30] were used depending on the most available data for each chemical of interest.

All analyses were performed using SAS software (SAS Institute Inc, Cary, NC, USA).

## Results

The geometric means and associated 95% CIs of detectable chemicals are listed in Table 1. Most phenols, parabens, and phthalates measured were detected in the urine samples. Conversely, only the metabolites for the TXIB alternative plasticizer, namely 2,2,4-trimethylpentane-1,3-diol (TMPD) and 2,2,4-trimethyl-3-hydroxyvaleric acid (HTMV), were detected.

BPA concentrations in Q2017 were over double the CHMS Cycle 6 (2018–2019) concentrations [geometric mean (95% confidence interval): 1.98 (1.69–2.31) versus 0.71 (0.60–0.84) µg/g creatinine] but were in line with FNBI (2011) [1.74 (1.41–2.13) µg/g creatinine]. Conversely, 2,4-dichlorophenol, 2,5-dichlorophenol, and triclosan concentrations in Q2017 were approximately half the concentrations in CHMS and FNBI. Concentrations of the remaining bisphenols were unavailable in CHMS and FNBI.

After stratifying by population characteristics, BPA did not differ by age, sex, or region (Table S2). Bisphenol-S concentrations were higher in females versus males [2.05 (1.50–2.81) versus 1.14 (0.93–1.39) µg/g creatinine], and 4,4’-biphenol concentrations were highest among those aged 40–59 years [1.68 (1.41–2.00)] versus those aged 16–19 years [1.07 (0.90–1.27)]. 2,4-Dichlorophenol concentrations were also higher in females [0.73 (0.57–0.93) versus 0.41 (0.27–0.60)], and 2,5-dichlorophenol was higher in Ungava Bay [5.73 (3.16–10.39)] versus the Hudson Bay [1.19 (0.83–1.69)].

Methylparaben concentrations in Q2017 were less than CHMS Cycle 6 (2018–2019) concentrations; however, ethylparaben was detected in 60.6% of Q2017 pooled samples compared to 36.1% of CHMS samples (Table 1). Comparisons of propylparaben concentrations could not be made with CHMS and FNBI due to unavailable or unreliable data. Paraben concentration differences by population characteristics were largely unreliable (Table S2).

Several urinary phthalate metabolites in Q2017 were higher to CHMS Cycle 6 (2018–2019) concentrations. MBzP, MCiNP, MCiOP, MHBP, MHiDP, MHiNP, MMP, and MOiDP were 1.5 to 2.5-fold higher in Q2017 versus CHMS Cycle 6 (2018–2019). MBzP, in particular, was 19-fold higher in Q2017 versus CHMS Cycle 6 (2018–2019) [45.26 (39.35–52.06) versus 2.4 (2.0–2.9) µg/g creatinine] and four-fold higher than in FNBI [18.58 (15.30–22.55) µg/g creatinine] (Table 1).

Although not always statistically significant, females generally had higher concentrations of all phthalates compared to men, particularly MBzP [52.45 (43.71–62.94) versus 38.83 (31.95–47.19) µg/g creatinine], MEP [38.47 (19.95–74.19) versus 12.81 (9.66–16.99) µg/g creatinine], MiBP [7.05 (6.33–7.85) versus 5.12 (4.55–5.76) µg/g creatinine], and 2-OH-MiBP [3.71 (3.22–4.29) versus 2.66 (2.35–3.02) µg/g creatinine] (Table S2).

MBzP and MHBP had higher levels in Hudson Bay [57.66 (46.82–71.02); 2.72 (2.47–3.01) µg/g creatinine] compared to Ungava Bay [35.22 (30.64–40.48); 2.02 (1.71–2.40) µg/g creatinine]. However, although not statistically significant, participants in Ungava Bay had generally higher concentrations of other phthalate metabolites including, MCiNP, MCiOP, MCPP, MEHP, MHiDP, MiBP, MMP, MOiNP, and 2-OH-MiBP. There were no differences in phthalate concentrations by age.

TXIB urinary metabolites, TMPD and HTMV, had four-fold higher concentrations in Q2017 compared to CHMS Cycle 6 (2018–2019) [TMPD: 52.33 (45.41–60.32) versus 13 (1116); HTMV: 9.16 (7.72–10.87) versus 2.4 (2.1–2.9) µg/g creatinine] (Table 1). Other plasticizer metabolites could not be compared to CHMS or FNBI concentrations; however, <50% of the CHMS population had detectable levels of OH-MINCH, oxo-MINCH, and *cis-cx-*MINCH, in contrast to the Q2017 population that had 93.2%, 81.8%, and 63.0% of detectable concentrations, respectively.

Although not statistically significant, females, older individuals, and residents of Ungava Bay communities had higher concentrations of TXIB metabolites (Table S2).

The following chemicals were detected in 0–14% of the pooled samples and were not further discussed in the results: phenols: pentachlorophenol, 2,4,6-trichlorophenol, 2,4,5-trichlorophenol; bisphenols: bisphenol-BPAF, bisphenol-AP, bisphenol-B, bisphenol-CII, phthalates: MCHP, MCHpP, MiDP, and MnOP; and alternative plasticizers: *cis*-CHDA, *trans*-MINCH, *trans*-cx-MINCH, 1-MEHTM, and 2-MEHTM.

## Discussion

This pilot study is the first to describe the biomarker concentrations of non-persistent chemicals in Nunavik. We detected higher concentrations of some non-persistent chemical biomarkers in Nunavik compared to CHMS, including BPA, certain phthalates, and TXIB metabolites (TMPD and HTMV). On the other hand, phenols, parabens and phthalates concentrations in Nunavik were generally lower than FNBI non-persistent chemical concentrations in 2011, with the exception of bisphenol A and MBzP. However, not all chemicals included in our study were available in CHMS and FNBI, so there may be other non-persistent chemicals of concern in our population. While some results were not statistically significant, women had generally higher concentrations of bisphenol-S, 2,4-DCP, 2,5-DCP, several phthalates (MBzP, MCiNP, MCiOP, MCPP, MECPP, MEHHP, MEHP, MEOHP, MEP, MHiDP, MiBP, Mnbp, MOiNP), and alternative plasticizer markers (TMPD, HTMV, OH-MINCH). The results also suggested higher levels of many of these chemicals in Ungava Bay.

BPA concentrations in Q2017 were similar to FNBI, but over double the CHMS Cycle 6 (2018–2019) and also higher than the concentrations reported in the U.S. National Health and Nutrition Examination Survey (NHANES) 2015–2016 [32], and the Maternal-Infant Research on Environmental Chemicals Study [34], a national-level prospective biomonitoring study carried out in pregnant women across Canada. Other bisphenols were not measured in CHMS and FNBI, but were included in NHANES 2013–2014 [32]. While bisphenol-F concentrations in Q2017 [0.82 (0.50–1.35) µg/g creatinine] were not significantly different than NHANES concentrations [0.53 (0.47–0.61) µg/g creatinine], bisphenol-S concentrations in Q2017 [1.53 (1.23–1.91) µg/g creatinine] were three-fold higher than NHANES concentrations [0.43 (0.39–0.47) µg/g creatinine]. The elevated concentrations of bisphenol-S and BPA may be concerning because bisphenol-S (a BPA alternative) is as hormonally active as BPA; and, bisphenol-S and bisphenol-F have been linked to endocrine-disrupting effects [35]. Diet is a major source of bisphenols, especially canned foods [36]. The elevated concentrations in Q2017 and elevated BPA concentrations in FNBI may be reflective of high consumption of canned foods in remote Indigenous communities, including those in the Arctic, since these can be easily cooked or transported on the land and due to the higher cost and poorer quality of perishable items [37].

Several phthalate compounds were higher in Q2017 compared to CHMS. MBzP in Q2017 was exceptionally high in Nunavik compared to CHMS and FNBI, and were also higher than concentrations in NHANES 2015–2016 [45.26 (39.35–52.06) versus 4.63 (4.06–5.28) µg/g creatinine] [32] and Alaskan Native women in 1999–2002 (7.1 µg/g creatinine) [23]. MBzP is a metabolite of butyl-benzyl phthalate, commonly used in vinyl flooring and detected in households since it is not covalently bound to the flooring [38, 39]. Vinyl is the most common flooring material used in Nunavik dwellings and many activities in Inuit homes take place sitting on the floor, including preparing/eating country foods and sewing, potentially contributing to the elevated concentrations. Other phthalate concentrations were likewise generally higher in Nunavik compared to concentrations reported in Greenland in 2002–2004 [22, 24]; but lower than concentrations measured in Alaskan Native women (with the exception of MBzP) in 1999–2002 [23]. No other recent studies measuring phthalate exposure in northern or Indigenous contexts were identified.

Similar to MBzP, TXIB is also commonly used in vinyl flooring [40, 41], and this could, again, explain the elevated concentrations in Nunavik versus CHMS in 2018–2019. TXIB has high vapor pressure and is a plasticizer with documented high emissions in air [10, 41]. A study in Japan analyzed indoor air samples from homes, and TXIB had the highest concentrations of the 59 compounds tested, whereas DINCH was undetected [42]. To our knowledge, no additional studies examined human exposure levels of TXIB.

The generally elevated concentrations of phenols and phthalates in women versus men in Q2017 was consistent with other studies in Norway [43], Belgium [44], China [45], and the U.S. [32], and this was attributed to the greater use of personal care products by women. Parabens were especially elevated in women versus men in these studies, but our paraben results were too unreliable to be compared by sex. Conversely, BPA had notably similar levels in men and women in Nunavik, and this was similar to the findings from CHMS Cycle 6 2018–2019 [30] and NHANES 2015–2016 [32]. These similar findings by sex are likely related to the similar patterns of use of plastic products.

We did not observe any clear differences in biomarker levels by age, although some phenol and phthalate concentrations increased with age and were slightly elevated among those aged 40–59 years. Middle-aged Nunavik Q2017 participants were also more likely to report consumption of market foods compared to other age groups [46]. As such, the higher concentrations could be indicative of their higher consumption of store-bought packaged foods (versus traditionally hunted or harvested foods) and consumer products. Interestingly, CHMS has shown levels of phenols, parabens, phthalates and alternative plasticizers are often highest in children aged 3–5 years and other younger age groups [30], and further studies are needed to document non-persistent chemical exposures and local exposure sources among younger Inuit populations in Nunavik and elsewhere in the Arctic.

Lastly, a study in Slovenia observed higher concentrations of phenols, parabens, and DINCH in industrialized versus rural communities [47]. Ungava Bay includes the town of Kuujjuaq, which is the largest village in Nunavik and the administrative capital of the Kativik Regional Government. Thus, participants living in Ungava Bay may have increased access to market foods and consumer products, explaining their elevated concentrations of some non-persistent chemicals, including dichlorophenols, some phthalates, and the alternative plasticizer, TXIB.

Our study had some limitations. Pooled samples are an effective method for biomonitoring and identification of more highly exposed groups [48], and allow for sufficient samples volumes for urinary analyses in a cost-effective manner. However, the statistical power is reduced using pooled samples compared to analyses using individual samples. We also had a limited number of pools, and outliers may have influenced the concentrations observed. However, we accounted for this by including CVs to test for dispersion. We were also unable to compare all the analyzed compounds to the general Canadian population and other Indigenous populations due to a lack of measurement of some compounds in CHMS and FNBI or due to a lack of reliability in the results. Larger studies are required to thoroughly confirm these findings, identify exposure sources, and assess their health impacts.

## Conclusion

We present a summary of the phenols, parabens, phthalates, and alternative plasticizers pooled sample results as a call to conduct further research in this area and protect the systematically and structurally excluded populations living in the Arctic. There is evidence of elevated concentrations of select bisphenols, phthalates, and alternative plasticizers that warrant further study, notably among women and Ungava Bay residents. The Inuit living in Nunavik are already exposed to high levels of persistent chemicals, and larger studies are required to confirm our findings, and assess the potential health impacts associated with these additional non-persistent chemical exposures.

## Supplementary material

Supplementary material
