# Supplementary material for: Non-persistent exposures from plasticizers or plastic constituents in remote Arctic communities: a case for further research
Source: J Expo Sci Environ Epidemiol. 2022 Mar 28;32(3):400–7. doi: 10.1038/s41370-022-00425-w (PMC9119853; doi:10.1038/s41370-022-00425-w)
Supplement: Supplementary file 1 — Supplementary material [file 41370_2022_425_MOESM1_ESM.pdf]

Supplementary material for “Non-persistent plasticizer exposures in remote Arctic communities: A case for further research”

Amira Aker[1,2], Élyse Caron-Beaudoin[3,4], Pierre Ayotte[1,2,5], Sylvie Ricard[6], Véronique Gilbert[7], Ellen Avard[8] and Mélanie Lemire[1,2]

1 Axe santé des populations et pratiques optimales en santé, Centre de recherche du CHU de Québec-Université Laval,1050 Ch Ste-Foy, Québec, QC G1S 4L8

2 Département de médecine sociale et préventive, Institut de biologie intégrative et des systèmes, Université Laval, 1030 Avenue de la Médecine, Québec, QC G1V 0A6

3 Department of Health and Society University of Toronto Scarborough, 1265 Military Trail, Toronto, ON, Canada

4 Centre for Clinical Epidemiology and Evaluation, University of British Columbia, Vancouver Coastal Health Research Institute, 828 West 10th Avenue, Research Pavilion, Vancouver, BC, Canada

5 Centre de Toxicologie du Québec, Institut National de Santé Publique du Québec, Québec, Canada

6 Nunavik Regional Board of Health and Social Services, Kuujjuaq, QC, Canada

7 Kativik Regional Government based in Saint-laurent, QC, Canada

8 Nunavik Research Centre, Makivik Corporation, Kuujjuaq, QC, Canada

Table S1: Pooled urinary sample descriptions in *Qanuilirpitaa? 2017*

Pool ID	Pool description			Number of individual samples per pool (n)
	Region	Sex	Age group	
1	Ungava Bay	M	16-19	27
2	Ungava Bay	M	20-29	38
3	Ungava Bay	M	30-39	29
4	Ungava Bay	M	40-59	54
5	Ungava Bay	M	60+	30
6	Ungava Bay	F	16-19	54
7	Ungava Bay	F	20-29	90
8	Ungava Bay	F	30-39	61
9	Ungava Bay	F	40-59	124
10	Ungava Bay	F	60+	39
11	Hudson Strait	M	16-19	22
12	Hudson Strait	M	20-29	12
13	Hudson Strait	M	30-39	8
14	Hudson Strait	M	40-59	32
15	Hudson Strait	M	60+	23
16	Hudson Strait	F	16-19	36
17	Hudson Strait	F	20-29	57
18	Hudson Strait	F	30-39	33
19	Hudson Strait	F	40-59	65
20	Hudson Strait	F	60+	16
21	Hudson Bay	M	16-19	39
22	Hudson Bay	M	20-29	37
23	Hudson Bay	M	30-39	30
24	Hudson Bay	M	40-59	54
25	Hudson Bay	M	60+	18
26	Hudson Bay	F	16-19	61
27	Hudson Bay	F	20-29	74
28	Hudson Bay	F	30-39	44
29	Hudson Bay	F	40-59	85
30	Hudson Bay	F	60+	34

Table S2: Geometric means of urine concentrations (µg/g creatinine) in 30 pooled samples created from the 1266 individuals recruited in Q2017 by sex, age, and region

Chemical	Pool	N	%≥LOD	GM (95% CI)
**PHENOLS**				
**Bisphenol A**	**Sex**			
	Females	15	100	1.99 (1.71 – 2.31)
	Males	15	100	1.96 (1.45 – 2.66)
	**Age**			
	16-19y	6	100	1.62 (1.35 – 1.96)
	20-39y	12	100	1.81 (1.49 – 2.20)
	40-59y	6	100	2.12 (1.62 – 2.77)
	60y+	6	100	[b]
	**Region**			
	Hudson Bay	10	100	1.99 (1.56 – 2.54)
	Hudson Strait	10	100	2.15[a] (1.20 – 3.84)
	Ungava Bay	10	100	1.84 (1.67 – 2.04)
**Bisphenol E**	**Sex**			
	Females	15	100	0.037 (0.035 – 0.040)
	Males	15	100	0.029 (0.026 – 0.032)
	**Age**			
	16-19y	6	100	0.034 (0.026 – 0.044)
	20-39y	12	100	0.035 (0.031 – 0.039)
	40-59y	6	100	0.031 (0.026 – 0.037)
	60y+	6	100	0.030 (0.020 – 0.045)
	**Region**			
	Hudson Bay	10	100	0.033 (0.029 – 0.038)
	Hudson Strait	10	100	0.034 (0.028 – 0.041)
	Ungava Bay	10	100	0.031 (0.027 – 0.036)
**Bisphenol F**	**Sex**			
	Females	15	100	0.80[a] (0.44 – 1.43)
	Males	15	100	[b]
	**Age**			
	16-19y	6	100	[b]
	20-39y	12	100	[b]
	40-59y	6	100	[b]
	60y+	6	100	0.33[a] (0.17 – 0.63)
	**Region**			
	Hudson Bay	10	100	0.50[a] (0.24 – 1.07)
	Hudson Strait	10	100	[b]
	Ungava Bay	10	100	[b]
**Bisphenol S**	**Sex**			
	Females	14	100	2.05 (1.50 – 2.81)
	Males	15	100	1.14 (0.93 – 1.39)
	**Age**			
	16-19y	6	100	1.14 (0.80 – 1.61)

Chemical	Pool	N	%≥LOD	GM (95% CI)
	20-39y	12	100	1.48 (1.10 – 1.97)
	40-59y	6	100	1.80[a] (0.93 – 3.51)
	60y+	5	100	[b]
	**Region**			
	Hudson Bay	10	100	1.50[a] (0.98 – 2.30)
	Hudson Strait	9	100	1.84[a] (1.01 – 3.37)
	Ungava Bay	10	100	1.38 (1.05 – 1.81)
**Bisphenol Z**	**Sex**			
	Females	15	100	0.033 (0.028 – 0.039)
	Males	15	92.1	0.027 (0.023 – 0.032)
	**Age**			
	16-19y	6	100	0.025 (0.021 – 0.030)
	20-39y	12	100	0.030 (0.027 – 0.033)
	40-59y	6	100	0.037 (0.030 – 0.047)
	60y+	6	63	0.020[a] (0.010 – 0.039)
	**Region**			
	Hudson Bay	10	95.9	0.030 (0.024 – 0.039)
	Hudson Strait	10	100	0.032 (0.027 – 0.037)
	Ungava Bay	10	93.5	0.028 (0.023 – 0.035)
**4,4'-Biphenol**	**Sex**			
	Females	15	100	1.30 (1.13 – 1.49)
	Males	15	100	1.21 (0.99 – 1.47)
	**Age**			
	16-19y	6	100	1.07 (0.90 – 1.27)
	20-39y	12	100	1.16 (1.03 – 1.31)
	40-59y	6	100	1.68 (1.41 – 2.00)
	60y+	6	100	0.95 (0.75 – 1.19)
	**Region**			
	Hudson Bay	10	100	1.27 (1.01 – 1.61)
	Hudson Strait	10	100	1.40 (1.17 – 1.68)
	Ungava Bay	10	100	1.13 (0.96 – 1.32)
**2,4-Dichlorophenol**	**Sex**			
	Females	15	100	0.73 (0.57 – 0.93)
	Males	15	86.8	0.41[a] (0.27 – 0.60)
	**Age**			
	16-19y	6	100	0.52[a] (0.34 – 0.80)
	20-39y	12	89.4	0.51[a] (0.31 – 0.84)
	40-59y	6	100	0.58[a] (0.36 – 0.94)
	60y+	6	83.2	[b]
	**Region**			
	Hudson Bay	10	84.9	0.50[a] (0.29 – 0.87)
	Hudson Strait	10	100	0.44 (0.31 – 0.64)
	Ungava Bay	10	100	0.71 (0.53 – 0.96)
**2,5-Dichlorophenol**	**Sex**			
	Females	15	100	3.12[a] (1.91 – 5.12)
	Males	15	100	2.33[a] (1.27 – 4.27)
	**Age**			

Chemical	Pool	N	%≥LOD	GM (95% CI)
	16-19y	6	100	[b]
	20-39y	12	100	2.01[a] (1.25 – 3.22)
	40-59y	6	100	[b]
	60y+	6	100	[b]
	**Region**			
	Hudson Bay	10	100	1.19 (0.83 – 1.69)
	Hudson Strait	10	100	4.27[a] (2.79 – 6.53)
	Ungava Bay	10	100	5.73[a] (3.16 – 10.39)
**Triclosan**	**Sex**			
	Females	15	100	34.06 (24.12 – 48.09)
	Males	15	77.8	[b]
	**Age**			
	16-19y	6	71.4	[b]
	20-39y	12	89.4	[b]
	40-59y	6	100	28.91[a] (12.74 – 65.57)
	60y+	6	83.2	[b]
	**Region**			
	Hudson Bay	10	84.9	[b]
	Hudson Strait	10	91.3	[b]
	Ungava Bay	10	93	21.04[a] (10.56 – 41.90)
**PARABENS**				
**Ethylparaben**	**Sex**			
	Females	15	77.8	[b]
	Males	15	42.8	–
	**Age**			
	16-19y	6	44	–
	20-39y	12	58.2	–
	40-59y	6	68.2	[b]
	60y+	6	74.1	[b]
	**Region**			
	Hudson Bay	10	56.7	–
	Hudson Strait	10	24.2	–
	Ungava Bay	10	93	[b]
**Methylparaben**	**Sex**			
	Females	15	100	[b]
	Males	15	90.4	[b]
	**Age**			
	16-19y	6	100	[b]
	20-39y	12	89.4	[b]
	40-59y	6	100	21.21[a] (9.76 – 46.09)
	60y+	6	100	[b]
	**Region**			
	Hudson Bay	10	89	[b]
	Hudson Strait	10	100	[b]
	Ungava Bay	10	100	26.40[a] (14.44 – 48.26)
**Propylparaben**	**Sex**			

Chemical	Pool	N	%≥LOD	GM (95% CI)
	Females	15	89	[b]
	Males	15	70.4	[b]
	**Age**			
	16-19y	6	58.8	–
	20-39y	12	69.1	[b]
	40-59y	6	100	[b]
	60y+	6	100	[b]
	**Region**			
	Hudson Bay	10	66.2	[b]
	Hudson Strait	10	77.1	[b]
	Ungava Bay	10	100	[b]
**PHTHALATES**				
**MBzP**	**Sex**			
	Females	15	100	52.45 (43.71 – 62.94)
	Males	15	100	38.83 (31.95 – 47.19)
	**Age**			
	16-19y	6	100	43.19 (38.04 – 49.04)
	20-39y	12	100	46.31 (34.72 – 61.77)
	40-59y	6	100	45.27 (32.91 – 62.26)
	60y+	6	100	43.97 (30.54 – 63.30)
	**Region**			
	Hudson Bay	10	100	57.66 (46.82 – 71.02)
	Hudson Strait	10	100	41.27 (31.72 – 53.69)
	Ungava Bay	10	100	35.22 (30.64 – 40.48)
**MCiNP**	**Sex**			
	Females	15	100	1.40[a] (0.85 – 2.32)
	Males	15	100	0.94 (0.66 – 1.34)
	**Age**			
	16-19y	6	100	0.94[a] (0.44 – 1.99)
	20-39y	12	100	1.13[a] (0.63 – 2.01)
	40-59y	6	100	1.25[a] (0.68 – 2.30)
	60y+	6	100	1.38[a] (0.62 – 3.09)
	**Region**			
	Hudson Bay	10	100	0.79[a] (0.54 – 1.16)
	Hudson Strait	10	100	[b]
	Ungava Bay	10	100	1.50 (1.05 – 2.14)
**MCiOP**	**Sex**			
	Females	15	100	2.07[a] (1.28 – 3.36)
	Males	15	100	1.35 (1.03 – 1.78)
	**Age**			
	16-19y	6	100	1.01[a] (0.63 – 1.62)
	20-39y	12	100	1.29 (1.01 – 1.65)
	40-59y	6	100	2.16[a] (1.20 – 3.88)
	60y+	6	100	[b]
	**Region**			
	Hudson Bay	10	100	1.34[a] (0.81 – 2.22)

Chemical	Pool	N	%≥LOD	GM (95% CI)
	Hudson Strait	10	100	1.93[a] (0.98 – 3.79)
	Ungava Bay	10	100	2.04[a] (1.39 – 3.01)
**MCMHP**	**Sex**			
	Females	15	100	[b]
	Males	15	100	1.17 (0.90 – 1.52)
	**Age**			
	16-19y	6	100	1.00 (0.67 – 1.48)
	20-39y	12	100	1.39 (0.97 – 1.99)
	40-59y	6	100	[b]
	60y+	6	100	1.73 (1.24 – 2.41)
	**Region**			
	Hudson Bay	10	100	1.04 (0.88 – 1.25)
	Hudson Strait	10	100	1.33 (1.12 – 1.58)
	Ungava Bay	10	100	[b]
**MCPP**	**Sex**			
	Females	15	100	0.80[a] (0.53 – 1.22)
	Males	15	100	0.59 (0.47 – 0.73)
	**Age**			
	16-19y	6	100	0.51[a] (0.27 – 0.98)
	20-39y	12	100	0.62 (0.51 – 0.74)
	40-59y	6	100	0.68[a] (0.39 – 1.19)
	60y+	6	100	[b]
	**Region**			
	Hudson Bay	10	100	0.57[a] (0.37 – 0.87)
	Hudson Strait	10	100	0.76[a] (0.45 – 1.27)
	Ungava Bay	10	100	0.83 (0.63 – 1.11)
**MECPP**	**Sex**			
	Females	15	100	8.21[a] (4.42 – 15.25)
	Males	15	100	4.69 (3.99 – 5.50)
	**Age**			
	16-19y	6	100	5.01 (3.92 – 6.40)
	20-39y	12	100	5.48 (4.53 – 6.63)
	40-59y	6	100	[b]
	60y+	6	100	6.85 (4.84 – 9.71)
	**Region**			
	Hudson Bay	10	100	4.78 (4.07 – 5.61)
	Hudson Strait	10	100	5.28 (4.62 – 6.04)
	Ungava Bay	10	100	[b]
**MEHHP**	**Sex**			
	Females	15	100	6.16[a] (3.70 – 10.24)
	Males	15	100	3.76 (3.26 – 4.33)
	**Age**			
	16-19y	6	100	3.77 (2.90 – 4.90)
	20-39y	12	100	4.38 (3.57 – 5.37)
	40-59y	6	100	[b]
	60y+	6	100	5.23 (3.70 – 7.40)
	**Region**			

Chemical	Pool	N	%≥LOD	GM (95% CI)
	Hudson Bay	10	100	3.85 (3.34 – 4.44)
	Hudson Strait	10	100	4.07 (3.45 – 4.81)
	Ungava Bay	10	100	[b]
**MEHP**	**Sex**			
	Females	15	100	1.23[a] (0.85 – 1.78)
	Males	15	100	0.88 (0.76 – 1.01)
	**Age**			
	16-19y	6	100	0.85 (0.63 – 1.16)
	20-39y	12	100	1.05 (0.95 – 1.15)
	40-59y	6	100	1.22[a] (0.52 – 2.85)
	60y+	6	100	0.90 (0.65 – 1.23)
	**Region**			
	Hudson Bay	10	100	0.90 (0.77 – 1.05)
	Hudson Strait	10	100	0.87 (0.76 – 1.01)
	Ungava Bay	10	100	1.45[a] (0.83 – 2.53)
**MEOHP**	**Sex**			
	Females	15	100	3.30[a] (1.93 – 5.65)
	Males	15	100	1.98 (1.72 – 2.28)
	**Age**			
	16-19y	6	100	1.99 (1.55 – 2.54)
	20-39y	12	100	2.35 (2.00 – 2.75)
	40-59y	6	100	[b]
	60y+	6	100	2.64 (1.83 – 3.81)
	**Region**			
	Hudson Bay	10	100	2.08 (1.79 – 2.42)
	Hudson Strait	10	100	2.15 (1.77 – 2.62)
	Ungava Bay	10	100	[b]
**MEP**	**Sex**			
	Females	15	100	38.47[a] (19.95 – 74.19)
	Males	15	100	12.81 (9.66 – 16.99)
	**Age**			
	16-19y	6	100	14.47 (9.58 – 21.84)
	20-39y	12	100	16.58[a] (10.05 – 27.35)
	40-59y	6	100	[b]
	60y+	6	100	[b]
	**Region**			
	Hudson Bay	10	100	[b]
	Hudson Strait	10	100	24.69[a] (11.86 – 51.42)
	Ungava Bay	10	100	22.98[a] (14.24 – 37.08)
**MHBP**	**Sex**			
	Females	15	100	2.45 (2.13 – 2.81)
	Males	15	100	2.10 (1.79 – 2.47)
	**Age**			
	16-19y	6	100	2.36 (2.09 – 2.67)
	20-39y	12	100	2.27 (1.83 – 2.80)
	40-59y	6	100	2.11 (1.70 – 2.61)
	60y+	6	100	2.67 (2.11 – 3.38)

Chemical	Pool	N	%≥LOD	GM (95% CI)
	**Region**			
	Hudson Bay	10	100	2.72 (2.47 – 3.01)
	Hudson Strait	10	100	1.92 (1.56 – 2.36)
	Ungava Bay	10	100	2.02 (1.71 – 2.40)
**MHiDP**	**Sex**			
	Females	15	100	0.64 (0.50 – 0.82)
	Males	15	100	0.60 (0.50 – 0.71)
	**Age**			
	16-19y	6	100	0.49 (0.36 – 0.65)
	20-39y	12	100	0.54 (0.43 – 0.69)
	40-59y	6	100	0.77 (0.63 – 0.95)
	60y+	6	100	0.81[a] (0.49 – 1.34)
	**Region**			
	Hudson Bay	10	100	0.70 (0.55 – 0.89)
	Hudson Strait	10	100	0.55 (0.43 – 0.72)
	Ungava Bay	10	100	0.56 (0.41 – 0.78)
**MHiDP**	**Sex**			
	Females	14	100	1.66[a] (1.02 – 2.72)
	Males	15	100	1.26 (0.90 – 1.76)
	**Age**			
	16-19y	6	100	0.97[a] (0.51 – 1.83)
	20-39y	11	100	1.19 (0.86 – 1.64)
	40-59y	6	100	1.57[a] (0.86 – 2.88)
	60y+	6	100	[b]
	**Region**			
	Hudson Bay	10	100	1.12[a] (0.68 – 1.84)
	Hudson Strait	9	100	1.32[a] (0.70 – 2.48)
	Ungava Bay	10	100	2.15[a] (1.46 – 3.17)
**MiBP**	**Sex**			
	Females	15	100	7.05 (6.33 – 7.85)
	Males	15	100	5.12 (4.55 – 5.76)
	**Age**			
	16-19y	15	100	5.73 (3.91 – 8.41)
	20-39y	15	100	5.64 (4.84 – 6.58)
	40-59y	15	100	6.25 (5.07 – 7.71)
	60y+	15	100	7.75 (6.00 – 10.00)
	**Region**			
	Hudson Bay	10	100	5.55 (4.78 – 6.45)
	Hudson Strait	10	100	6.13 (4.91 – 7.65)
	Ungava Bay	10	100	6.64 (5.50 – 8.00)
**MiNP**	**Sex**			
	Females	15	71.4	0.34[a] (0.22 – 0.51)
	Males	15	50.9	–
	**Age**			
	16-19y	6	47.5	–
	20-39y	12	75.2	0.34[a] (0.23 – 0.51)
	40-59y	6	46.2	–

Chemical	Pool	N	%≥LOD	GM (95% CI)
	60y+	6	65.8	[b]
	**Region**			
	Hudson Bay	10	37.5	–
	Hudson Strait	10	51.7	–
	Ungava Bay	10	100	0.44 (0.36 – 0.55)
**MMP**	**Sex**			
	Females	15	100	2.17 (1.85 – 2.54)
	Males	15	100	1.99 (1.41 – 2.81)
	**Age**			
	16-19y	6	100	2.11 (1.44 – 3.10)
	20-39y	12	100	1.88 (1.55 – 2.29)
	40-59y	6	100	2.47[a] (1.28 – 4.77)
	60y+	6	100	1.90[a] (1.15 – 3.14)
	**Region**			
	Hudson Bay	10	100	1.87 (1.43 – 2.44)
	Hudson Strait	10	100	2.26[a] (1.26 – 4.04)
	Ungava Bay	10	100	2.25 (1.86 – 2.74)
**MnBP**	**Sex**			
	Females	15	100	15.96 (14.02 – 18.16)
	Males	15	100	12.42 (10.61 – 14.55)
	**Age**			
	16-19y	6	100	14.11 (12.13 – 16.40)
	20-39y	12	100	13.78 (10.89 – 17.44)
	40-59y	6	100	13.89 (11.56 – 16.69)
	60y+	6	100	16.36 (12.55 – 21.33)
	**Region**			
	Hudson Bay	10	100	16.76 (14.55 – 19.29)
	Hudson Strait	10	100	11.77 (9.40 – 14.73)
	Ungava Bay	10	100	12.89 (10.99 – 15.11)
**MOiDP**	**Sex**			
	Females	15	100	0.56 (0.47 – 0.67)
	Males	15	100	0.53 (0.43 – 0.64)
	**Age**			
	16-19y	6	100	0.52 (0.43 – 0.63)
	20-39y	12	100	0.49 (0.39 – 0.62)
	40-59y	6	100	0.57 (0.45 – 0.72)
	60y+	6	100	0.79[a] (0.50 – 1.24)
	**Region**			
	Hudson Bay	10	100	0.62 (0.49 – 0.79)
	Hudson Strait	10	100	0.50 (0.40 – 0.62)
	Ungava Bay	10	100	0.48 (0.39 – 0.59)
**MOiNP**	**Sex**			
	Females	11	100	1.00[a] (0.62 – 1.62)
	Males	13	100	0.69 (0.49 – 0.97)
	**Age**			
	16-19y	4	100	0.69[a] (0.36 – 1.32)
	20-39y	8	100	0.78 (0.55 – 1.12)

Chemical	Pool	N	%≥LOD	GM (95% CI)
	40-59y	6	100	0.68 (0.45 – 1.02)
	60y+	6	100	[b]
	**Region**			
	Hudson Bay	8	100	0.79[a] (0.45 – 1.40)
	Hudson Strait	7	100	[b]
	Ungava Bay	9	100	0.95 (0.70 – 1.29)
**2-OH-MiBP**	**Sex**			
	Females	15	100	3.71 (3.22 – 4.29)
	Males	15	100	2.66 (2.35 – 3.02)
	**Age**			
	16-19y	6	100	3.01 (2.21 – 4.10)
	20-39y	12	100	2.83 (2.35 – 3.40)
	40-59y	6	100	3.47 (2.71 – 4.44)
	60y+	6	100	4.13 (3.38 – 5.04)
	**Region**			
	Hudson Bay	10	100	2.85 (2.43 – 3.34)
	Hudson Strait	10	100	3.30 (2.61 – 4.18)
	Ungava Bay	10	100	3.48 (2.74 – 4.43)
**ALTERNATIVE PLASTICIZERS**				
**TXIB Metabolites**				
**TMPD**	**Sex**			
	Females	15	100	59.11 (47.12 – 74.16)
	Males	15	100	46.11 (38.85 – 54.73)
	**Age**			
	16-19y	6	100	42.71 (30.19 – 60.42)
	20-39y	12	100	55.08 (42.53 – 71.33)
	40-59y	6	100	57.27 (43.74 – 74.99)
	60y+	6	100	44.01[a] (24.72 – 78.37)
	**Region**			
	Hudson Bay	10	100	50.17 (38.59 – 65.22)
	Hudson Strait	10	100	51.34 (36.11 – 73.00)
	Ungava Bay	10	100	56.14 (43.85 – 71.87)
**HTMV**	**Sex**			
	Females	15	100	10.25 (7.83 – 13.42)
	Males	15	100	8.15 (6.50 – 10.22)
	**Age**			
	16-19y	6	100	6.99[a] (4.56 – 10.72)
	20-39y	12	100	9.78 (7.15 – 13.39)
	40-59y	6	100	10.64 (7.90 – 14.33)
	60y+	6	100	6.73[a] (3.97 – 11.43)
	**Region**			
	Hudson Bay	10	100	8.38 (6.17 – 11.38)
	Hudson Strait	10	100	8.74 (6.40 – 11.95)
	Ungava Bay	10	100	10.66 (7.47 – 15.20)
**DINCH Metabolites**				
**OH-MINCH**	**Sex**			

Chemical	Pool	N	%≥LOD	GM (95% CI)
	Females	15	95.5	0.17 (0.13 – 0.22)
	Males	15	90.8	0.10 (0.07 – 0.14)
	**Age**			
	16-19y	6	86.2	0.11[a] (0.05 – 0.24)
	20-39y	12	94.7	0.13 (0.09 – 0.19)
	40-59y	6	100	0.15[a] (0.09 – 0.23)
	60y+	6	78.1	0.13[a] (0.06 – 0.27)
	**Region**			
	Hudson Bay	10	94.7	0.13 (0.09 – 0.18)
	Hudson Strait	10	81.5	0.12[a] (0.07 – 0.22)
	Ungava Bay	10	100	0.15[a] (0.10 – 0.22)
**oxo-MINCH**	**Sex**			
	Females	15	92.4	0.056 (0.044 – 0.070)
	Males	15	70.9	0.030[a] (0.019 – 0.046)
	**Age**			
	16-19y	6	86.2	0.037[a] (0.019 – 0.074)
	20-39y	12	84.4	0.044[a] (0.027 – 0.073)
	40-59y	6	88.3	0.044[a] (0.024 – 0.079)
	60y+	6	46.2	–
	**Region**			
	Hudson Bay	10	90.6	0.048 (0.035 – 0.066)
	Hudson Strait	10	67.3	0.029[a] (0.015 – 0.055)
	Ungava Bay	10	81.2	0.043[a] (0.023 – 0.079)
**cis-CX-MINCH**	**Sex**			
	Females	15	86.7	0.045 (0.037 – 0.055)
	Males	15	40.1	–
	**Age**			
	16-19y	6	71.4	[b]
	20-39y	12	67.3	0.032[a] (0.021 – 0.049)
	40-59y	6	68.2	0.034[a] (0.018 – 0.065)
	60y+	6	25.9	–
	**Region**			
	Hudson Bay	10	65.8	0.033[a] (0.021 – 0.052)
	Hudson Strait	10	67.3	0.032[a] (0.019 – 0.053)
	Ungava Bay	10	58.8	–

If >40% of samples were below the LOD, the percentile distribution is reported but means were not calculated.
a Use data with caution. The coefficients of variation is between 16.6% and 33.3%.
b Data is too unreliable to be published. The coefficients of variation is greater than 33.3%.

Table S3: Limits of detection for non-persistent chemicals included in study

Chemical of Interest	Limit of detection (LOD) (µg/L)
ENVIRONMENTAL PHENOLS AND TRICLOSAN	
Bisphenol A (BPA)	0.018
Bisphenol AF (BPAF)	0.025
Bisphenol AP (BPAP)	0.11
Bisphenol B (BPB)	0.0039
Bisphenol CII (BPC II)	0.022
Bisphenol E (BPE)	0.0061
Bisphenol F (BPF)	0.094
Bisphenol S (BPS)	0.092
Bisphenol Z (BPZ)	0.024
Bisphenol 4,4'	0.076
Triclosan	4.4
PARABENS	
Butylparaben (BPB)	0.3
Ethylparaben (EPB)	0.9
Methylparaben (MPB)	1
n-Propylparaben (PPB)	0.3
PHTHALATE METABOLITES	
Monobenzyl phthalate (MBzP)	0.37
Monocyclohexyl phthalate (MCHP)	0.25
Mono-(7-carboxy-n-heptyl) phthalate (MCHpP)	0.073
Mono(carboxy-isononyl) phthalate (MCiNP)	0.077
Mono-(carboxy-isooctyl) phthalate (MCiOP)	0.30
Mono-(2-carboxy-methylhexyl) phthalate (MCMHP)	0.18
Mono-3-carboxypropyl phthalate (MCPP)	0.12
Mono-(2-ethyl-5-carboxypentyl) phthalate (MECPP)	0.20
Mono-(2-ethyl-5-hydroxyhexyl) phthalate (MEHHP)	0.19
Monoethylhexyl phthalate (MEHP)	0.11
Mono-(2-ethyl-5-oxohexyl) phthalate (MEOHP)	0.088
Monoethyl phthalate (MEP)	0.98
Mono-(3-hydroxy-n-butyl) phthalate (MHBP)	0.079
Mono(hydroxy-isodecyl) phthalate (MHiDP)	0.067
Mono(hydroxyl-isononyl) phthalate (MHiNP)	0.056
Mono-iso-butyl phthalate (MiBP)	0.13
Monoisodecyl phthalate (MiDP)	0.16
Monoisononyl phthalate (MiNP)	0.37
Monomethyl phthalate (MMP)	0.16
Mono-*n*-butyl phthalate (MnBP)	0.39
Mono-*n*-octyl phthalate (MnOP)	0.15
Monooxoisodecyl phthalate (MOiDP)	0.070
Monooxoisononyl phthalate (MOiNP)	0.085
Mono-2-hydroxy-isobutyl phthalate (2OH-MiBP)	

ALTERNATE PLASTICIZERS AND THEIR METABOLITES	
2,2,4-Trimethyl-1,3-pentanediol (TMPD)	1.7
2,2,4-Trimethyl-3-hydroxy valeric acid (HTMV)	0.42
Cyclohexane-1,2-dicarboxylic acid (cis-CHDA)	0.30
1,2-(*trans*-Cyclohexane-dicarboxylate)-mono-4-methyloctyl ester (*trans*-MINCH)	0.010
1,2-(Cyclohexanedicarboxylate)-mono-(7-hydroxy-4-methyl) octyl ester (OH-MINCH)	0.078
1,2-(Cyclohexane-dicarboxylate)-mono-(7-oxo-4-methyl) octyl ester (oxo-MINCH)	0.029
1,2-(*trans*-Cyclohexane-dicarboxylate)-mono-(7-carboxylate-4-methyl)heptyl ester (*trans*-cx-MINCH)	0.28
1,2-(*cis*-Cyclohexane-dicarboxylate)-mono-(7-carboxylate-4-methyl) heptyl ester (*cis*-cx-MINCH)	0.042
1,2,4-Benzenetricarboxylate 1-(2-ethylhexyl) ester (1-MEHTM)	0.15
1,2,4-Benzenetricarboxylate 2-(2-ethylhexyl) ester (2-MEHTM)	0.11
1,2,4-Benzenetricarboxylate 4-(2-ethylhexyl) ester (4-MEHTM)	0.081
